# Body oscillations couple with wing flapping to reduce aerodynamic power in wild silk moth flight

**DOI:** 10.1098/rsif.2025.0061

**Published:** 2025-08-27

**Authors:** Usama Bin Sikandar, Brett R. Aiello, Simon Sponberg

**Affiliations:** ^1^ School of Electrical and Computer Engineering, Georgia Institute of Technology, Atlanta, GA 30322, USA; ^2^ School of Physics, Georgia Institute of Technology, Atlanta, GA 30322, USA; ^3^ Department of Biology, Seton Hill University, Greensburg, PA, USA; ^4^ School of Biological Sciences, Georgia Institute of Technology, Atlanta, GA 30332, USA

**Keywords:** flight, aerodynamics, insect, blade-element model, wings, flapping

## Abstract

Insects show diverse flight kinematics and morphologies reflecting their evolutionary histories and ecological adaptations. Many silk moths use low wingbeat frequencies and large wings to fly and display body oscillations. Their bodies pitch and bob periodically, synchronized with their wingbeat cycle. Similar oscillations in butterflies improve weight support and forward thrust while reducing flight power requirements. However, how instantaneous body and wing kinematics interact for these beneficial aerodynamic and power consequences is not well understood. We hypothesized that body oscillations affect aerodynamic power requirements by influencing wing rotation relative to the airflow. Using three-dimensional forward flight video recordings of four silk moth species and a quasi-steady blade-element aerodynamic model, we analysed the aerodynamic effects of body and wing kinematics. We find that the body pitch and wing sweep angles maintain a narrow range of phase differences, which enhances the angle of attack variation between each half-stroke due to increased wing rotation relative to the airflow. This redirects the aerodynamic force to increase the upward and forward components during the downstroke and upstroke, respectively, thus lowering overall drag without compromising weight support and forward thrust. Reducing energy expenditure is beneficial because many adult silk moths do not feed and rely on limited energy budgets.

## 1. Introduction

The incredible variation of insect morphology and flight behaviours is a key factor in their wide diversification. One of the most ubiquitous flight behaviours is forward flight, which insects use to explore, forage, find mates and migrate. To vary their forward speed, insects such as hawkmoths, bees and flies primarily vary their body pitch angles to tilt their stroke planes. This orients the wingstroke-averaged aerodynamic force at an angle relative to gravity that enables forward flight while providing sufficient weight support [1]. By holding the body at a constant angle, they can rotate their wings around the hinge (a motion known as feathering or wing pitching) to precisely adjust the angle of attack and redirect aerodynamic force into useful directions throughout the wingstroke. However, in many forward-flying butterflies and silk moths, the body pitch angle has been observed to continuously and periodically vary during a wingstroke [2–4]. This periodic body rotation adds to the wing rotation relative to the incoming airflow, in addition to the periodic rotation from wing pitching about the thorax, leading to an oscillating stroke plane. As a result, the body’s pitch oscillations supplement the wing’s own rotation, allowing butterflies and silk moths to achieve the necessary overall wing rotation relative to the air with relatively small wing rotations about the thorax. Smaller wing rotations in butterflies have been shown to improve wake capture and enhance forward thrust [5]. Moreover, butterflies and silk moths have also been observed to bob during each wingstroke [3,4,6].

The superimposition of body pitching and bobbing oscillations onto the wing’s pitching about the thorax adds complexity to flight control, as it requires simultaneously tuning the angle of attack and redirecting aerodynamic forces to propel the body forward and support its weight. These body oscillations arise from inherent wing–body coupling dynamics present in all flapping insects, but they are especially pronounced in butterflies with low wing loading and wingbeat frequency [7,8]. Similar coupling may occur in silk moths with large wings and low wingbeat frequencies [4]. However, the precise association between the magnitude of body oscillations and parameters such as wingbeat frequency, wing loading and wing flapping amplitude remains unclear, despite these factors influencing both aerodynamic and inertial forces. Thus, it is non-trivial which of these forces—or both—primarily drives the body oscillations, and whether these oscillations are subject to independent control through reconfiguration of the thorax, abdomen and wings. Such inertial reconfiguration allows rotations in diverse organisms, from lizards to cats to dinosaurs [9,10] and has been explicitly shown to provide a mechanism for control in hawkmoths, the evolutionary cousins of silk moths [11,12]. Similar cases in biomechanics exist where active movement of one body part induces passive oscillations in another, influencing the overall energy requirements of locomotion. For example, in humans, passive arm swinging coupled with walking reduces metabolic energy requirements by 12% compared with steady volitional arm holding, and by 26% compared with antiphase arm swinging [13].

Regardless of whether body oscillations occur passively or are actively generated, we can investigate their consequences for aerodynamic force and power production. Many silk moths face tight energy constraints as they do not feed as adults. This presents a conundrum: body oscillations probably increase energy expenditure by adding to the kinetic cost of motion. Do these insects use strategies to conserve power during flight? This question motivates us to explore how aerodynamic power requirements would change in the absence of body oscillations.

In this study on silk moth flight, we aim to answer four questions to present a more holistic understanding of the aerodynamic and power implications of body oscillations. First, we investigate which wing movements and morphological parameters are associated with the magnitude of body oscillations in the kinematic modelling fits of four silk moth species. This analysis is meant to establish exemplars with different degrees of body oscillations and wing kinematics rather than to specifically compare the full-flight kinematics of the four species. Second, using a quasi-steady blade-element method, we investigate whether the inertial force or the aerodynamic force is the main driver of the silk moth body oscillations through wing–body coupling. In birds, the generation of body oscillations is dominated by inertial forces from wing flapping [14]. However, butterfly studies show that body oscillation can be explained by the influence of flapping-wing aerodynamics on the body [7]. Third, we explore the aerodynamic consequences of body oscillations, given the pattern of the species-averaged wing and body kinematics observed. We analyse how observed body oscillations affect the wing angle of attack, angle of inclination and relative airflow speed. The angle of attack and relative airflow speed prescribe the aerodynamic force, while the angle of inclination specifies the direction of the force. These quantities must be controlled to ensure a successful flight. Fourth, we explore how the coupling between body oscillations and flapping-wing motion influences flight performance—specifically in terms of weight support, forward thrust and aerodynamic power. We ask whether a preferred phase difference between body oscillations and wing motion exists that enhances performance across these parameters, or whether such coupling, regardless of phase, leads to reduced performance compared with a hypothetical body dynamics model with no body oscillations.

## 2. Methods

### 2.1. Body and wing measurements and morphometrics

In this study, we used live specimens of four available wild silk moth species that occupy a wide range of wingbeat frequencies and wing sizes. The four species were *Automeris io* (three specimens and four wingstrokes), *Actias luna* (three specimens and one wingstroke each), *Antheraea polyphemus* (two specimens and one wingstroke each) and *Hyalophora euryalus* (one specimen and four wingstrokes). Body and wing morphologies were digitized for each live specimen using our previous imaging techniques [4] using StereoMorph (v. 1.6.2) [15] in R software (v. 3.4.2). After each moth specimen had flown, its total body mass ($m_{t}$) was recorded. The morphological data were subsequently processed in MATLAB (v. R2018b-9.5.0.944444) as per [4,16]. To create a combined wing shape from the intersection of the forewings and hindwings, we adjusted the forewing’s long axis to make it perpendicular to the body’s long axis. The hindwing was kept in its natural position, the state it is in when the wings are spread wide and the moth is idle. This orientation was chosen after examining silk moth flight videos in which the hindwing’s long axis is consistently oriented posteriorly and parallel to the body’s long axis [4]. The representative shape and size of the wings of each species are shown in electronic supplementary material, figure S1, along with the position of the wing hinge and pitching axis. All wing morphological parameters used for aerodynamic analysis were derived from this combined wing, as described by [17]. All morphological parameter values of the individual moth specimens are provided in electronic supplementary material, table S1.

### 2.2. Moth free-flight high-speed recordings

Live specimens were filmed in a wind tunnel where they flew at forward speeds between 2 and 3 m s${,}^{-1}$. We conducted free-flight experiments inside a 100 × 60.96 cm segment of an open-circuit Eiffel-style wind tunnel provided by ELD, Inc. from Lake City, MN, USA. For a detailed description of the tunnel, see [18]. The moths were prompted to fly at a wind speed of 0.7 m s${,}^{-1}$. We captured silk moth flight sequences at 1000 frames s${,}^{-1}$ using three synchronized high-speed cameras (resolution: 1280 × 1024 pixels), specifically the Mini UX 100 from Photron, San Diego, CA, USA. The lighting in the wind tunnel consisted of six 850 nm infrared lamps from Larson Electronics, Kemp, TX, USA, alongside a neutral density-filtered light-emitting diode (LED) ‘moonlight’ (Neewer CN-126) [19]. The footage was then processed and standardized in XMALab [20]. We identified and marked seven critical points on each flying moth specimen, namely the rostral tip of the head (situated between the antennae), the thorax–abdomen intersection, the rear end of the abdomen, the right and left forewing hinges (attachment points to the thorax), the tip of the right wing and the point at the inner angle of the right forewing (intersection point of the forewing and hindwing on the distal edge of the wing). These seven points were identifiable and thus could be consistently tracked across species.

### 2.3. The quasi-steady aerodynamic model

**Figure 1.**
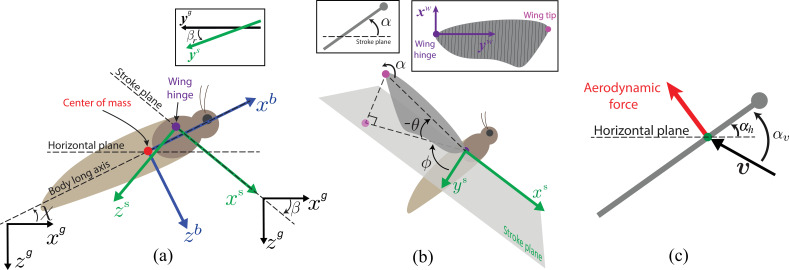
(a–c) Illustrate the coordinate systems and kinematic parameters used in the modelling and analysis (adapted from [4]). (a) The global (inertial) frame $Ox^{g}y^{g}z^{g}$, the body-attached frame $Ox^{b}y^{b}z^{b}$ and the stroke-plane frame $Ox^{s}y^{s}z^{s}$. The origin of the body-attached frame is the centre-of-mass point, marked in red. The origin of the stroke-plane frame is the wing hinge point, marked in purple. The body (pitch) angle χ is the angle the body-long axis makes with the (absolute) horizontal plane $x^{g}y^{g}$ (the plane orthogonal to the direction of gravity), stroke-plane angle β is the angle the stroke-plane makes with the horizontal plane $x^{g}y^{g}$ and stroke-plane roll angle $\beta_{r}$ is the angle that $y^{s}$-axis makes with the $y^{g}$-axis (see inset). (b) Definitions of wing kinematic angles. The wing sweep angle ϕ and the wing deviation angle θ are the azimuthal and the elevation angles of the wing tip in the stroke-plane frame. The wing-pitching angle α (shown in the inset on the left) is the angle that the wing plane makes with the stroke plane. The leading edge of the wing is marked with a filled circle. Since the wing is assumed to be a flat plate without any bending or torsion, α is constant along the wing. The inset on the right shows the right wing plane with the wing-attached coordinate frame $Ox^{w}y^{w}z^{w}$, chord-wise elemental strips of the blade-element model and wing hinge and wing tip points. (c) Definitions of relative airflow velocity v, angle of attack $\alpha_{v}$ and wing inclination angle $\alpha_{h}$ as they are related to a wing strip in the blade-element model. The airflow relative to a wing strip makes an angle $\alpha_{v}$ with it while the strip is inclined at an angle $\alpha_{h}$ relative to the horizontal plane $x^{g}y^{g}$. Because α is constant along the wing, $\alpha_{h}$ is also constant.

All the kinematic measurements and force calculations we performed are represented in one of these four coordinate frames (figure 1): the global (inertial) frame $Ox^{\text{g}}y^{\text{g}}z^{\text{g}}$, body frame (attached to the body) $Ox^{\text{b}}y^{\text{b}}z^{\text{b}}$, stroke-plane frame $Ox^{\text{s}}y^{\text{s}}z^{\text{s}}$ and wing frame (attached to the wing) $Ox^{\text{w}}y^{\text{w}}z^{\text{w}}$. The time series of the seven tracked points in three dimensions was used to extract three-dimensional body velocity components (u, v and w in the global frame), body pitch angle χ (figure 1a) and wing kinematic parameters using custom MATLAB code as previously performed in [16]. In figure 1a,b, the wing kinematic parameters used to specify the wing motion were the wing kinematic angles including the sweep angle ϕ, the deviation angle θ and the feathering angle α (wing-pitching angle about $y^{\text{w}}$ axis), the stroke-plane angle β (figure 1a)—a convention similar to [21]—and the stroke-plane roll angle $\beta_{r}$—the angle that $y^{s}$-axis makes with the $y^{g}$-axis. When estimating the orientation of the stroke plane per wingstroke, we observed that the stroke plane did not incline only at an angle β relative to the absolute horizontal. It also underwent a slight rotation about the stroke-plane x-axis, which introduced a minor tilt $\beta_{r}$ in the direction of the resulting aerodynamic force on each wing. However, during steady forward flight with symmetric kinematics between the left and right wings, the aerodynamic effects of $\beta_{r}$ on each wing are equal in magnitude but opposite in direction and thus cancel each other out, although they still produce small aerodynamic effects on the individual wings.

The moths were recorded in steady flight, and thus we expect the wing and body kinematics to be periodic. We extracted the time series of each wing and body kinematic parameter and curve-fit each wingstroke with a periodic function that sufficiently captured the waveform shape. A third-order Fourier series consistently provided the best fits across the entire dataset. The Fourier series for ϕ is provided below as an example.


(2.1) $$\begin{array}{lcr} & \phi (t)=a_{\phi ,0}+\sum_{k=1}^{3}a_{\phi ,k}\cos(2\pi kft)+b_{\phi ,k}\sin(2\pi kft), & \end{array}$$

where $a_{\phi ,k}$ and $b_{\phi ,k}$ are Fourier series coefficients of the kth harmonic, t is the time measured in seconds and f is the wingbeat frequency. By averaging wing shapes and Fourier-fitted time-dependent kinematics across all wingstrokes for each individual moth, we obtained a representative set of total mass, wing shape, size and kinematic parameters for each species. The values of the individual and the averaged parameters are given in the electronic supplementary material, tables S12. It is important to note that the mean and amplitude values of the representative kinematic waveforms are not used directly in our calculations. As detailed in §2.4, the representative waveforms serve only to capture the shape of the motion, while the actual parameter values are determined through a trim search constrained by experimentally observed minimum and maximum bounds for each wingstroke parameter (table 1). We then used the ‘trimmed’ parameters to calculate species-specific aerodynamic forces using our previously published quasi-steady blade-element model [4]. This model calculates the aerodynamic force considering the forces from translational and rotational movements of the wing [16,22], as well as the force resulting from the added mass [16,23]. Translational aerodynamic forces were calculated using lift and drag coefficient functions of the angle of attack, $\alpha_{\text{v}}$, derived in previous studies from experiments on dynamically scaled wings of the hawkmoth *Manduca sexta* [24]. To compute the aerodynamic forces, each wing was divided into 200 chord-wise strips, and the translational, rotational and added-mass components of the aerodynamic force were calculated for each strip over a wingstroke divided into 1000 equal time steps. The equations governing the forces acting on a small wing strip of width dr are given below.

**Table 1.** Set of trim search bounds and a sample characteristic trim solution. Minimum (min.) and maximum (max.) values represent the extrema of each parameter observed in the experimental data of individual free-flight trials. The ‘trim’ values represent a characteristic trim solution set chosen from the 10 trim search results given in the electronic supplementary material, table S3. The same trim solution set is shown as solid curves in figure 2. The wingbeat frequency *f* is in Hz and the angles are in degrees. The scaling parameters *kL* and *kD* are dimensionless. Note that a few trim values fall slightly outside the specified minimum–maximum bounds, as the bounds were allowed to relax within a small threshold.

	*A. io*			*A. luna*			*A. polyphemus*			*H. euryalus*		
	min.	max.	trim	min.	max.	trim	min.	max.	trim	min.	max.	trim
f	20	24	21.938	13	15	13.011	7	13	8.872	12	14	12.329
	2	24	22.818	−4	27	26.946	18	21	20.016	34	53	48.363
	7	17	12.768	20	27	23.637	37	51	43.718	15	25	22.428
	36	76	60.748	66	82	68.876	54	68	64.007	22	60	44.245
$\beta_{a}$	7	18	7.592	20	26	26.002	32	57	38.995	14	28	25.858
	0	4	1.461	−1	2	−0.99	−5	1	−1.388	−2	1	−1.378
$\bar{\phi }$	5	44	37.293	7	17	16.424	26	38	33.461	5	40	31.608
	103	149	127.09	124	134	124.076	119	148	123.126	100	127	100.296
$\bar{\alpha }$	76	88	82.674	78	85	85.019	82	89	85.871	82	93	89.384
	43	71	55.389	32	44	43.99	45	94	45.67	29	51	36.175
$\bar{\theta }$	−1	1	0.322	0	1	0.171	−1	0	−0.528	−1	1	−0.15
	13	22	17.838	21	29	21.019	14	38	30.98	12	33	20.715
$k_{L}$	0.5	2	1.705	0.5	2	1.295	0.5	2	1.128	0.5	2	1.383
	0.6	1.4	0.6	0.6	1.4	0.6	0.6	1.4	0.609	0.6	1.4	0.612


(2.2)dFLw=12ρCLv2c dr F^Lw ,(2.3)dFDw=12ρCDv2c dr F^Dw ,(2.4)dFrotw=ρCRvc2α˙h dr F^rotw ,(2.5)dFadmw=14πρ((ϕ¨sin⁡α+ϕ˙α˙hcos⁡α)rc2+14α¨hc3)dr F^admw ,

where $F_{\text{L}}^{\text{w}}$ and $F_{\text{D}}^{\text{w}}$ are the lift and drag components of the translational aerodynamic force (aerodynamic force arising from translation of the wing relative to the thorax), $F_{\text{rot}}^{\text{w}}$ is the rotational aerodynamic force (force arising from circulation due to wing rotation relative to the thorax), $F_{\text{adm}}^{\text{w}}$ is the force due to added mass (aerodynamic force required to accelerate the added-mass of the air in the boundary layer of a wing), ρ is the air density, v is the relative airflow speed on the strip calculated as the speed of the strip measured in the global frame (assuming no wind condition), dr is the fixed width of a wing strip, and ${\hat{F}}_{\text{L}}^{\text{w}}$, ${\hat{F}}_{\text{D}}^{\text{w}}$, ${\hat{F}}_{\text{rot}}^{\text{w}}$ and ${\hat{F}}_{\text{adm}}^{\text{w}}$ denote unit vectors in the direction of the corresponding force components. Drag points in the direction of the relative airflow velocity v (opposite to the instantaneous velocity of the strip in the global frame), lift is perpendicular to v, and rotational and added-mass forces are perpendicular to the surface of the strip (see [4] for specific details). The lift and drag coefficients $C_{\text{L}}$ and $C_{\text{D}}$, respectively, are time-varying functions of the angle of attack $\alpha_{\text{v}}$ given by $C_{\text{L}}=1.552\;\sin\;\alpha_{\text{v}}\cos\;\alpha_{\text{v}}+1.725\;\sin^{2}\;\alpha_{\text{v}}\cos\;\alpha_{\text{v}}$, $C_{\text{D}}=0.0596\;\sin\;\alpha_{\text{v}}\cos\;\alpha_{\text{v}}+3.598\;\sin^{3}\;\alpha_{\text{v}}$ [24]. The theoretical value of the rotational aerodynamic coefficient $C_{\text{R}}=\pi \left(0.75-\frac{e}{c}\right)$ is based on the two boundary conditions of zero fluid flow across the wing surface and zero vorticity generated by the trailing edge element i.e., Kutta condition [25], where e is the position of wing-pitching axis relative to the leading edge of the wing, c is the chord length (varies for each strip and is determined by wing shape). Other parameters in equations (2.2)–(2.5) include the wing inclination angle $\alpha_{\text{h}}$, which is the angle of inclination of wing surface relative to the absolute horizontal plane (figure 1), where $\alpha_{\text{h}}\approx \alpha -\beta$ and its derivative represents the angular velocity of the wing-pitching rotation in the global frame [1,26], α is the wing feathering angle and β is the stroke-plane angle. The angle of attack for a wing strip (shown in figure 1c) is calculated as $\alpha_{\text{v}}=\cos^{-1}(-\hat{b}.\hat{v})$, where $\hat{b}$ is the unit vector along the chord line from the trailing edge of the strip to its leading edge, and $\hat{v}$ is the unit vector of the relative airflow velocity on the strip. Each force on a small wing strip of width dr in equations (2.2)–(2.5) is integrated across the entire wing to calculate the total force on the wing. We calculated the total aerodynamic force on both wings by first adding the translational, rotational and aerodynamic components on each wing in the wing frame, then transforming the total force on each wing to the body frame, and then adding together the left and right wing forces to yield the total aerodynamic force $F_{\text{aer}}^{\text{b}}$ from both wings. The aerodynamic force is then transformed to the global frame ($F_{\text{aer}}^{\text{g}}$) to analyse forward thrust and weight support using fore–aft ($x^{\text{g}}$) and vertical ($z^{\text{g}}$) components.

A detailed formulation of our blade-element model is provided in [4]. This model, which uses closed-form expressions, offers the flexibility to directly examine how different combinations of wing shapes and kinematics influence aerodynamic force and power across a range of species. It also enables us to investigate how individual force components (translational, rotational and added-mass) contribute to the overall aerodynamic force profile.

Because the wings and body are mechanically connected, inertial forces generated by wing flapping act on the moth’s body alongside aerodynamic forces, influencing its instantaneous motion in the air. To assess the relative contribution of these inertial effects, we calculated the reaction force on the body resulting from wing acceleration and compared it with the total aerodynamic force. This analysis quantifies the magnitude of the inertial force and evaluates its role in driving body oscillations. Measured in the global frame, the inertial force experienced by the body due to a wing strip of width dr moving at a velocity $v^{\text{g}}$ in the global frame is


(2.6) $$\begin{array}{rl} dF_{\text{inr}}^{\text{g}} & =-\frac{m_{\text{w}}}{S}c\;dr\frac{d}{dt}\left(v^{\text{g}}\right), \end{array}$$

where $m_{\text{w}}$ is the mass of the wing, S is the area of the wing and c is the chord length of the strip. Similarly, this equation was numerically integrated across all wing strips to calculate the total inertial force of a wing. Inertial forces are internal to the moth’s anatomical parts, and thus do not generate a net force during steady wingstrokes.

However, aerodynamic forces may be indirectly influenced by wing inertial forces (and the associated torques) through their effect on body movement. That said, because wing kinematics were measured relative to a fixed stroke plane relative to the body, and all forces were transformed into the global frame, any relative motion between the wings and body caused by inertial forces, along with its aerodynamic consequences, is inherently accounted for in the analysis.

In addition to forces, we also calculated aerodynamic power on the wings to determine the effect of body oscillations on the energy consumption of wing–airflow interactions. The instantaneous aerodynamic power dissipated on a small wing strip of width dr is the work done by aerodynamic force per unit time and is given by the following equation:


(2.7) $$dP_{\text{aer}}=(dF_{\text{D}}+dF_{\text{rot}}+dF_{\text{adm}}).\;v,$$

where $dF_{\text{D}}$ is the instantaneous drag force, $dF_{\text{rot}}$ is the instantaneous rotational aerodynamic force and $dF_{\text{adm}}$ is the instantaneous force due to added mass on the wing strip and v is the airflow velocity incident on the wing strip. The lift $dF_{\text{L}}$ is not part of the power calculation because it is perpendicular to v and thus does not contribute to the aerodynamic power. The drag on the body (parasitic drag) has also not been included because it is small compared with the drag on the wings [4]. The total power $P_{\text{aer}}$ was calculated by numerically integrating equation (2.7) and adding together the power contribution of both wings.

### 2.4. Trim search

The kinematics of the free-flying moths that we measured did not produce steady-state or balanced aerodynamic forces and torques from our blade-element model, despite periodic and nearly steady wingstrokes. In addition, the fact that the wingstrokes were not perfectly steady could also be due to measurement or modelling errors. For example, we modelled the wings as rigid flat plates, which may result in small errors in the aerodynamic force calculation because the wings can bend slightly while flapping. Furthermore, the lift and drag coefficients in our model were based on experimental studies on the hawkmoth *M. Sexta* rather than the silk moths in this study. Therefore, the values of not only the aerodynamic coefficients but also the wing kinematic parameters may require slight adjustments based on similar precedents set in other studies [27]. Thus, to achieve steady-state flight in the model prior to analysis, we performed a trim search on the wing and body kinematics and aerodynamic parameters, similar to our method in [4]. We essentially searched (inside a bounded space) for the values of kinematic and aerodynamic parameters that created equilibrium in forces and moments averaged over a wingstroke. The search space was bounded by the natural ranges of parameters that we measured in our experiments (table 1). The objective of the trim search was to ensure the following three equilibrium conditions on the wingstroke-averaged force and moment components: ${\bar{F}}_{x}^{\text{g}}=0,{\bar{F}}_{z}^{\text{g}}=mg,{\bar{M}}_{y}^{\text{g}}=0$. Other force and moment components $F_{y}$, $M_{x}$ and $M_{z}$ were zero due to the assumption of steady forward flight in which the left and right wing flapping motions are perfectly identical, and body movement or turning in the lateral plane are essentially zero (no body translation along the $y^{b}$-axis and no body rotation about the $x^{b}$- or $z^{b}$-axis).

In our model, some parameters are held constant during a wingstroke, while others vary. The wingbeat frequency f and the stroke-plane roll angle $\beta_{\text{r}}$ remain constant throughout each wingstroke. In contrast, the wing kinematic angles ϕ, θ and α, along with the stroke-plane angle β and body pitching angle χ, vary over the course of a wingstroke. However, to perform the trim search, we needed to identify features of these time-varying parameters that could be held fixed during a single trim search iteration. Specifically, we selected the mean values and amplitudes of the waveforms for ϕ, θ, α, β and χ to remain constant over one flapping cycle. The mean is computed as the arithmetic average of the sampled values of each parameter across a wingstroke, while the amplitude is defined as the difference between the maximum and minimum values in the waveform. The complete set of kinematic parameters included in the trim search space (table 1) comprises: wingbeat frequency f; stroke-plane roll angle $\beta_{\text{r}}$; body pitch angle amplitude $\chi_{a}$ and mean χ (where the subscript ‘a’ denotes waveform amplitude and the overline indicates mean value); stroke-plane angle amplitude $\beta_{a}$ and mean β; wing sweep angle amplitude $\phi_{a}$ and mean ϕ; wing feathering angle amplitude $\alpha_{a}$ and mean α; wing deviation angle amplitude $\theta_{a}$ and mean θ; and the aerodynamic coefficient scaling factors $k_{\text{D}}$ and $k_{\text{L}}$ applied to the drag and lift coefficients $C_{\text{D}}$ and $C_{\text{L}}$, respectively. This variation in aerodynamic coefficients accounts for interspecific differences in wing morphology and slight changes in flight conditions.

We set up the trim search as a minimization problem on the cost function,


(2.8) $$\begin{array}{lcr} & G={\left(\frac{{\bar{F}}_{x}^{\text{g}}}{mg}\right)}^{2}+{\left(\frac{{\bar{F}}_{z}^{\text{g}}}{mg}-1\right)}^{2}+{\left(\frac{{\bar{M}}_{y}^{\text{g}}}{mgr_{2}}\right)}^{2}, & \end{array}$$

which must be zero at perfect trim equilibrium. To ensure dimensional consistency and appropriate scaling, the force and moment values are normalized, respectively, with weight mg, and the product of weight mg and radius $r_{2}$ of the second moment of area of a wing. During minimization of G, the trim search parameters were bounded between the minimum and maximum values that we measured across all of our recorded wingstrokes of the species in question (table 1). For all species, we also constrained the parameters $k_{\text{D}}$ and $k_{\text{L}}$ within the ranges (table 1) based on the measured variation in the mean lift and drag coefficients in [28] for flight conditions comparable to our data. Bounding the search space ensures that the trimmed aerodynamics still correspond to the respective natural range of kinematics of each species. Within the search space, we solved for zero-value local minima of the cost function G using an open-source MATLAB function fminsearchbnd() [29], which takes the cost function, initial condition and bounding region in the search space as input arguments, and returns the values of the function and coordinates of the search space at a local minimum.

It is important to recognize that the search space for minimizing the cost function G is defined by a nonlinear aerodynamic model and therefore may contain multiple local and global minima. As a result, a particular parameter combination identified through the trim search is not guaranteed to be unique or correspond to a global minimum. To address this, we performed multiple trim search runs, each initialized with randomized parameter values within the defined search space bounds, and continued this process until 10 distinct global minimum solutions were obtained for each silk moth steady flight condition.

**Figure 2.**
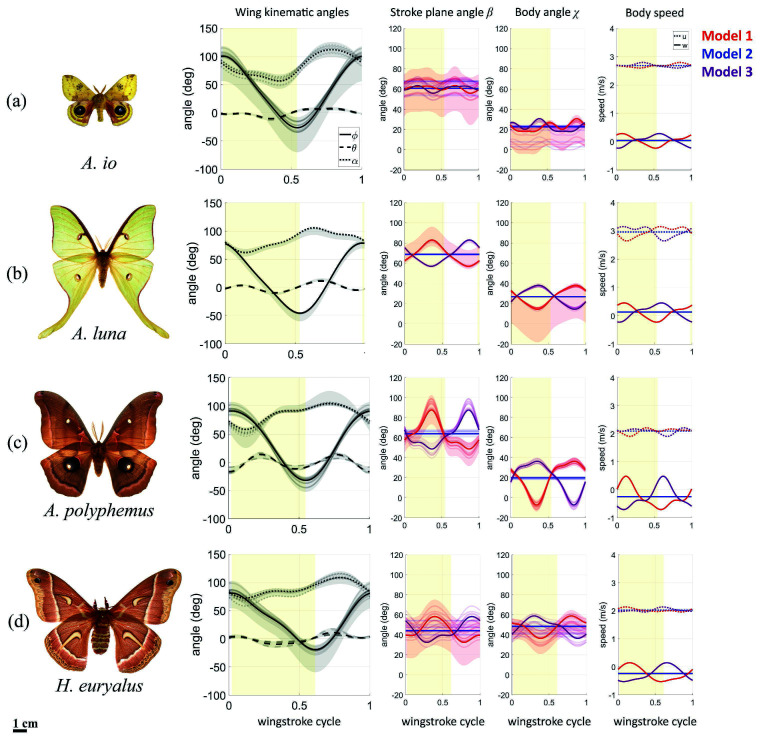
Panels a–d showing the wing kinematic angles and the three model configurations (Models 1, 2 and 3) of body kinematics of the four silk moth species used in this study: (a) *A. io*, (b) *A. luna*, (c) *A. polyphemus* and (d) *H. euryalus* (some data re-used from [4]). The yellow-shaded part of roughly half of each wingstroke cycle represents a downstroke and the remaining part is the upstroke. The solid curves in the plots of wing kinematic angles, stroke-plane angle β and body angle χ represent one characteristic trim solution from a total of 10 solutions for each species. The transparent curves represent the remaining nine trim solutions. The shaded regions represent the trim search space bounded by observed extrema of kinematic parameters in our experimental data. The first column of plots shows wing kinematic angles ϕ, θ and α, which capture the wing movement relative to the stroke plane, with the stroke-plane angle β shown in the second column. The third and fourth columns show important body kinematic parameters (body pitch angle χ, body velocity fore–aft u and vertical w components) plotted in the three model configurations. Model 1 (red) represents the time-varying configuration of body kinematics. Model 2 (blue) represents the wingstroke-averaged version of body kinematics. Model 3 (purple) has Model 1 kinematics shifted by a half wingstroke period to introduce a $180^{\circ }$ phase shift relative to the flapping-wing motion.

A set of characteristic trim conditions found for each species is given in table 1, while the full set of 10 trim conditions is detailed in electronic supplementary material, table S3. The kinematics plots in figure 2 also show the individual trim solutions and the search bounds. All sets of trim solutions are close to each other in the search space. The existence of multiple solutions suggests the presence of multiple, yet similar and proximate, kinematic strategies for the same steady flight condition, as observed in previous experimental studies [21].

### 2.5. Comparative models of body kinematic configuration

To assess the effects of body oscillations and their coupling with wing flapping on the aerodynamic performance of silk moth flight, we used three kinematic model configurations, shown in figure 2, to calculate aerodynamic forces and power, while keeping the wing kinematic angles fixed. Model 1 represents the experimentally measured time-varying kinematics. Model 2 represents hypothetical kinematics with no body oscillations, where body kinematics are fixed at their wingstroke-averaged values, i.e. the wingstroke-averaged values of stroke-plane angle (β), body pitch angle (χ) and fore–aft (u) and vertical (w) components of the body velocity. Model 3 has the same time-varying body kinematics as Model 1, but shifted in time by a half period, and is also a hypothetical scenario to introduce a $180^{\circ }$ phase shift relative to the wing kinematics. In other words, Model 3 body kinematics are antiphase to the real flight in Model 1. Across the three models, the wing kinematic angles used are the same time-varying periodic functions shown in figure 2 for each species. These model configurations are useful because the aerodynamic differences between Models 1 and 2 isolate the impact of body oscillations, i.e. body oscillations versus no body oscillations. The differences between Models 1 and 3 reveal the effect on flight performance of the natural phase difference between the wing and body kinematics, as observed in steady forward flight.

## 3. Results

### 3.1. The amplitude of body oscillations varies with wingbeat frequency and wing loading

**Figure 3.**
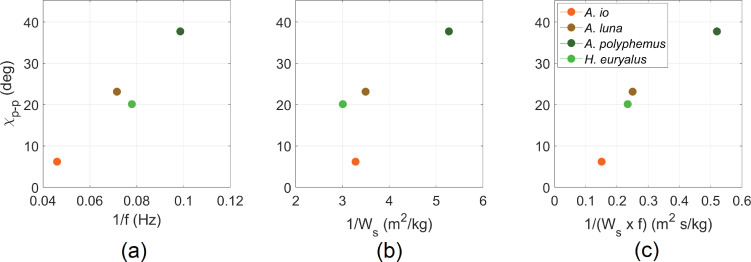
Scatter plots showing how the amplitude of body angle χ is associated with (a) wingbeat frequency (f), (b) wing loading $W_{s}$ and (*c*) the product of f and $W_{s}$. There is a correlation in (a) between the amplitude of χ and wingbeat time-period (1/f): r=0.97,p<0.05. There is also a correlation in (b) between the amplitude of χ and $W_{s}$, r=−0.79,p>0.05. Overall, a correlation exists between the amplitude of χ and the inverse of the product of $W_{s}$ and f: r=0.94,p<0.05.

First, we explored the morphological and wing kinematic parameters that underlie the variation in the magnitude of the body oscillation. We plotted the oscillation amplitudes of the body pitch angle χ and the body vertical velocity w against several morphological and wing kinematic parameters. Silk moth *A. io* displayed noticeably smaller χ oscillation amplitude compared with the other three species, with an amplitude of $6.9^{\circ }$. However, for the other three species, the χ amplitude ranged between $21.8^{\circ }$ and $38.5^{\circ }$. The w oscillation amplitude was also the lowest for *A. io* at 0.5 m s${,}^{-1}$, while the w amplitude for other species ranged between 0.67 and 1.19 m s${,}^{-1}$. Across the four silk moth species, we found that χ amplitude is correlated with wingbeat frequency f (figure 3a) and the product of f and wing loading ($W_{s}$) (figure 3c), where wing loading is defined as the total wing area divided by the total mass (wings and body). This means that silk moths with lower f, smaller body mass and larger wings have larger χ oscillations. However, extrapolating from this small dataset to all silk moths requires a more extensive comparative study. Our goal here is to explore the aerodynamic consequences of variation in bobbing (w) and pitching (χ) oscillations. The species we sampled show substantial variation in these parameters, which justifies their use as exemplars for our study.

### 3.2. Both aerodynamic and inertial forces contribute to body oscillations in silk moths

**Figure 4.**
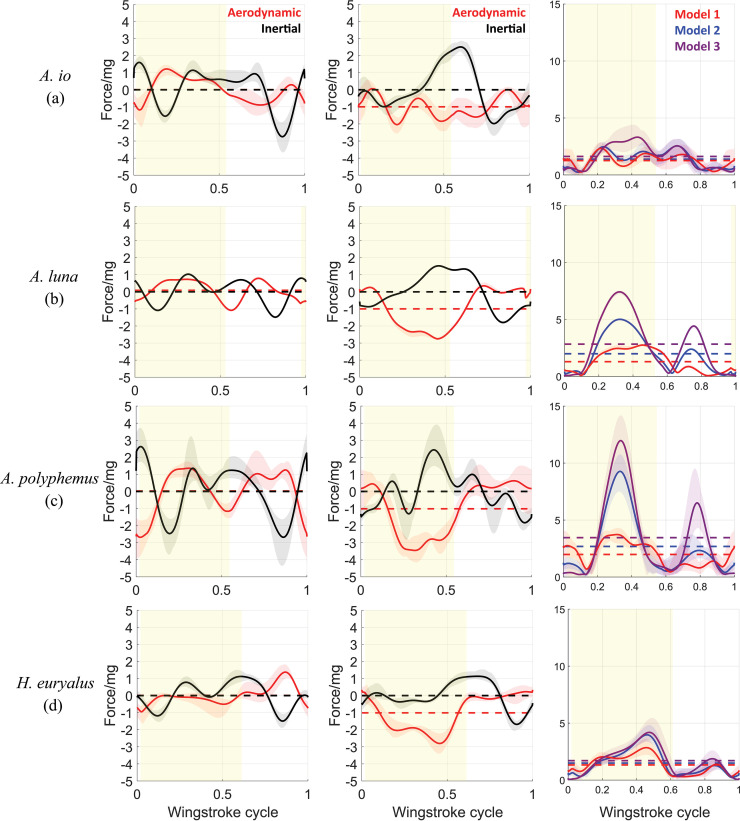
Panels a*–*d show the normalized x and z components (global frame) of aerodynamic and inertial forces in the first and second columns, and the total aerodynamic force in the third column generated from trimmed kinematics for the four silk moth species: (a) *A. io*, (b) *A. luna*, (c) *A. polyphemus* and (d) *H. euryalus*. The shaded regions around curves show the full variation of the 10 trim solutions found between the trim search bounds. Solid curves represent the mean curves averaged across the 10 trim solutions in each case. The normalization makes the forces physically equivalent to non-dimensional acceleration as multiples of g (gravitational acceleration). Right column shows the total aerodynamic force for each species generated from Model 1 (red), Model 2 (blue) and Model 3 (purple) configurations of wing kinematics. The yellow-shaded part of roughly half of each wingstroke cycle represents a downstroke, and the remaining part represents the upstroke.

Using these four exemplar species, we next investigated how body oscillations relate to the forces generated by wing flapping. We applied our quasi-steady blade-element method to determine whether aerodynamic or inertial forces from the wings primarily drive body oscillations. To this end, we compared the normalized aerodynamic and inertial forces (figure 4) generated using the trimmed flapping-wing motion and body kinematics from Model 1 (figure 2). Comparing the shapes and magnitudes of the two waveforms, we find that aerodynamic and inertial forces have comparable magnitudes, and thus both forces contribute to silk moth body oscillations. Therefore, in contrast to some bird flight studies where inertial forces dominate [14], and butterfly studies where aerodynamic forces are the primary driver of body oscillations [7], we found that aerodynamic and inertial forces in silk moths are comparable in magnitude and contribute roughly equally to driving body oscillations.

### 3.3. Body oscillations affect aerodynamic force magnitude and direction

Next, we determined the aerodynamic consequences of body oscillations in body pitch angle χ and body speed components u and w. A kinematic variation in any one of these parameters can potentially affect the magnitude as well as the direction of the aerodynamic force. To analyse these effects, we plot the total aerodynamic force corresponding to Model 1 (with body oscillations) and Model 2 (without body oscillations). The plots of silk moths *A.luna*., *A.polyphemus*. and *H.euryalus*. (figure 4b–d column 3) show large differences in the mean (up to 1.8×) and peak (up to 3×) aerodynamic force magnitudes between Models 1 and 2. In the absence of body oscillations (Model 2), the magnitude of aerodynamic forces would have been greater during most of the wingstroke, resulting in higher mean and peak force values.

**Figure 5.**
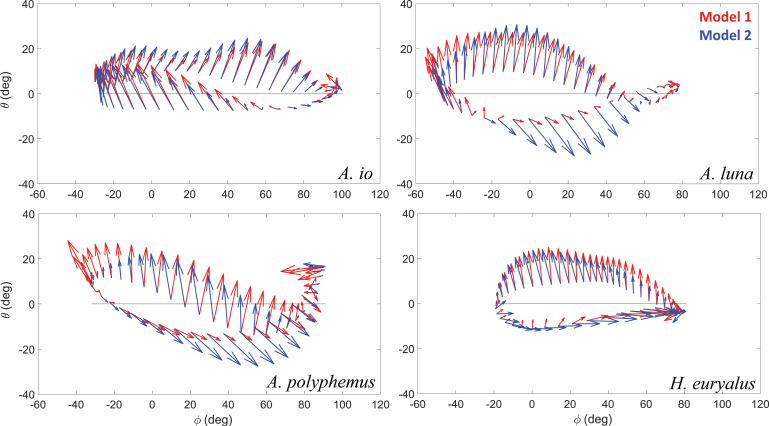
Arrows showing direction and relative magnitude of the total aerodynamic force corresponding to one characteristic trimmed kinematics set of each species (a) *A. io* (b) *A. luna* (c) *A. polyphemus* (d) *H. euryalus* for Model 1 (red) and Model 2 (blue). In each case, the direction of flight is to the right. Roughly, the arrows above the black horizontal line correspond to the downstroke, and the ones below correspond to the upstroke. The ends represent the stroke reversals.

The direction of the aerodynamic force between Models 1 and 2 also shows considerable differences and sometimes prominent switches (figure 5). During the Model 1 downstroke, *A.luna*. and *A.polyphemus*. aerodynamic force not only provides weight support but also a small forward thrust, directing them in usable directions. The aerodynamic force of the silk moth *H.eeuryalus*. during the downstroke in Model 1 is directed more upward compared with Model 2, and with a smaller backward component. This means that Model 1 kinematics provides more force for weight support than backward thrust. Compared with these three silk moths, a difference in strategy was observed in the case of silk moth *A.io*, which experiences smaller body oscillations. Although all four silk moths produced weight support in the downstroke, *A.io*. also produced small weight support in the upstroke. Whereas *A.luna*, *A.polyphemus*. and *H.euryalus*. produced downward force or a negligible upward force, slightly negating the overall weight support. But for these three silk moth species, which experience large body oscillations, this difference brings about an enhanced forward thrust during the upstroke because, for most of the upstroke, a significant component of the aerodynamic force is directed forward. Comparing Models 1 and 2 for these three species during the upstroke, the aerodynamic force in Model 1 is directed more forward and less downward than in Model 2. This shift enhances forward thrust while reducing the negative impact on overall weight support. Thus, these differences indicate that due to large body oscillations, the aerodynamic force requirement is reduced by directing the force components in usable directions that support body weight and generate forward thrust.

### 3.4. Sources of the effects on aerodynamic force magnitude and direction

**Figure 6.**
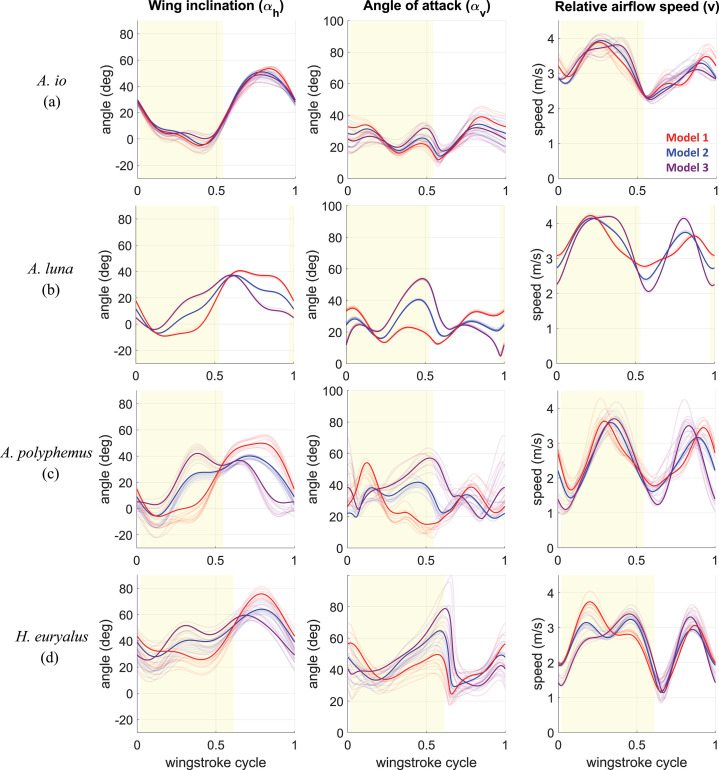
Graphs showing measured waveforms of wing inclination angle $\alpha_{h}$, wing's mean (averaged across all blade-element strips) angle of attack $\alpha_{v}$, and relative airflow speed v for each species: (a) *A. io*, (b) *A. luna*, (c) *A. polyphemus* and (d) *H. euryalus*. Solid curves represent one characteristic trim solution set for each species out of 10 trim solutions. The remaining nine solutions are shown as transparent curves. Model 1 (red) represents the time-varying configuration of body kinematics. Model 2 (blue) is the wingstroke-averaged version of body kinematics. Model 3 (purple) is Model 1 kinematics shifted by a half period to introduce a 180${,}^{\circ }$ phase shift relative to flapping-wing motion. The yellow-shaded part of roughly half of each wingstroke cycle represents a downstroke, and the remaining part is the upstroke.

Large body oscillations in silk moths *A.luna*., *A.polyphemus*. and *H.euryalus*. reduce the magnitude of the total aerodynamic force and redirect it into more functionally useful directions during each half-stroke, prompting us to examine which kinematic parameters contribute to these aerodynamic effects. There are three determinants of aerodynamic effects from the wing–air interaction: Wing inclination angle $\alpha_{h}$, wing angle of attack $\alpha_{v}$ and relative airflow speed v. The plots of how each of these varies in Models 1 and 2 are shown in figure 6.

#### 3.4.1. $\alpha_{h}$ Primarily influences the direction of the aerodynamic force

The wing inclination angle $\alpha_{h}$ is the key determinant of the direction of the aerodynamic force during both half-strokes because the aerodynamic force is roughly perpendicular to the wing surface, opposite to the surface experiencing airflow. There are noticeable differences in the waveforms of the wing inclination angle $\alpha_{h}$ between Models 1 and 2 in *A.luna*., *A.polyphemus*. and *H.euryalus*. (figure 6). During the mid-half of the downstroke, the variation in $\alpha_{h}$ is minimal. This is because, first, during the second quarter of the downstroke, the body pitches (χ) nose-down, while the wing-pitch rotation (α) about the thorax is in the opposite direction (figure 1). Thus, the χ oscillation roughly cancels the overall wing rotation in the global coordinate frame. Second, during the third quarter of the downstroke, the variation in both χ and α is negligible. Both of these kinematic features combine to hold the $\alpha_{h}$ waveform flat at a small value (up to $30^{\circ }$ lower than Model 2) for most of the middle part of the downstroke. This keeps the wing surface nearly horizontal, holding the aerodynamic force more vertical (upward), thus providing better weight support over most of the downstroke than in Model 2. During the mid-upstroke, once again, the variation in χ either counters α rotation (figure 1 *A.luna*.) or is as slow as α rotation (figure 1 *A.polyphemus*. and *H.euryalus*.), thus holding $\alpha_{h}$ at a higher value for a longer time. This keeps the wing surface at a high $\alpha_{h}$ (up to $15^{\circ }$ higher than Model 2), thus providing forward thrust for most of the upstroke. This is reflected in the form of a flight strategy in which the downstroke is dominated by generating weight support, while the upstroke is dominated by generating forward thrust. In the absence of body oscillations, the moth will either lose the ability to hold the wing inclination in preferred orientations or will need to actively hold it during both half-strokes. Overall, despite continuous active wing rotation around the thorax, the body pitch oscillations help hold the wings at functionally useful orientations over most of each half-stroke, thus ensuring that aerodynamic force stays in useful directions for a longer time. This essentially improves the flight strategy of prioritizing weight-support generation during the downstroke and forward thrust generation during the upstroke.

#### 3.4.2. $\alpha_{\text{v}}$ Primarily influences the magnitude of the aerodynamic force

The aerodynamic force magnitude differences between Models 1 and 2 are primarily driven by differences in the angle of attack ($\alpha_{v}$) when the relative airflow speed (v) is high (figure 6). This is mostly the case in the second half of the downstroke, and thus, we also see corresponding large differences in aerodynamic force magnitude between Models 1 and 2 (figure 4). During the middle part of the downstroke, particularly for *A.luna*. and *A.polyphemus*., $\alpha_{v}$ stays roughly between $10^{\circ }$ and $30^{\circ }$ in Model 1, with slight variation due to the wing inclination being held almost constant as described earlier. Operating at these smaller angles for a longer time when the relative airflow speed is high allows sufficient generation of lift force for flight while keeping the drag force low, thus resulting in a high lift-to-drag ratio and more efficient flight. On the other hand, in Model 2, without body oscillation, the angle of attack has a larger peak that increases to $50^{\circ }$, where the drag would increase significantly, resulting in a reduced lift-to-drag ratio. In *H.euryalus*., Model 2 results in a higher drag throughout most of the second half of the downstroke. Thus, in the presence of body oscillations in Model 1, the flight is still more efficient due to a higher lift-to-drag ratio during the portion of the wingstroke where most of the force is generated, i.e. the middle part of the downstroke.

### 3.5. Kinematic sources of $\alpha_{v}$ differences between Models 1 and 2

**Figure 7.**
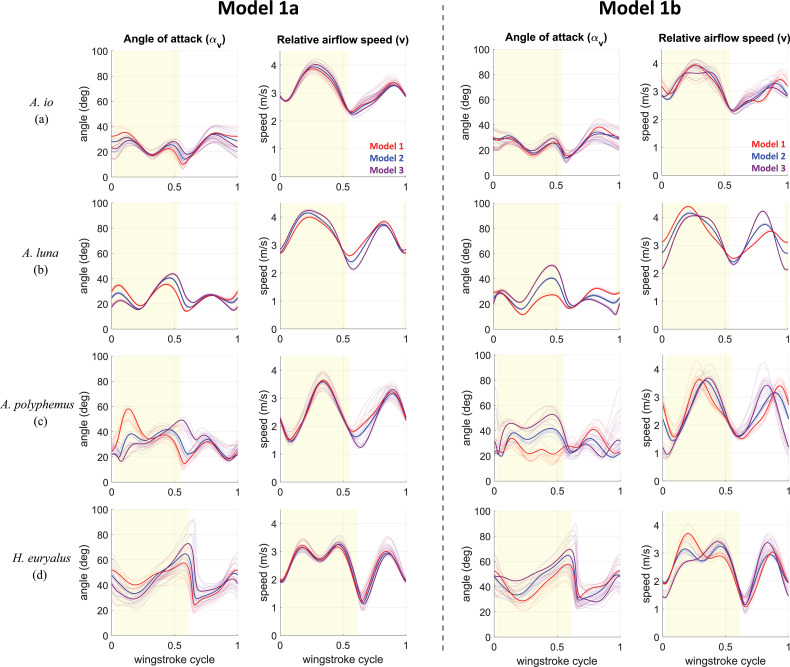
Kinematic configuration Model 1a (Model 1 without body pitch χ oscillations) and Model 1b (Model 1 without body speed u and w oscillations). Graphs showing waveforms at trim of wing inclination angle $\alpha_{\text{h}}$, wing's mean (strip-averaged) angle of attack $\alpha_{\text{v}}$ and relative airflow speed v for each species: (a) *A. io*, (b) *A. luna*, (c) *A. polyphemus* and (d) *H. euryalus*. The solid curves represent one characteristic trim solution set for each species out of 10 trim solutions. The remaining nine solutions are the transparent curves. The yellow-shaded part of roughly half of each wingstroke cycle represents the downstroke, and the remaining part is the upstroke.

In steady forward flight, wing inclination ($\alpha_{h}$) is purely driven by changes in the wing-pitch (α) and body-pitch (χ) angles and is not affected by the body velocity components (u and w). However, to determine the effect of χ, u and w on the wing angle of attack ($\alpha_{v}$), that is, precisely which of these drives the variation in $\alpha_{v}$, we simulated intermediate hypothetical models of Models 1 and 2. Intermediate Model 1a includes body speed (u and w) oscillations but no body pitch (χ) oscillations (left half of figure 7), while Model 1b has χ oscillations but no u or w oscillations (right half of figure 7).

The $\alpha_{v}$ differences between Models 1 and 2 throughout the wingstroke are generally driven by oscillations in both body speed (Model 1a) and χ (Model 1b). During the first half of the downstroke, body speed oscillations contribute the most to $\alpha_{v}$ differences. However, in the second half, the χ oscillations drive the $\alpha_{v}$ difference, particularly in silk moths *A.luna*. and *A.polyphemus*., which were flying with relatively larger χ amplitude but smaller χ values on average (larger stroke-plane angle β).

Near stroke reversals, relative airflow speed differences are caused by body speed oscillations, while mid-half-stroke differences are driven by χ oscillations (figure 7). This is expected, as the wing translational speed is minimal near stroke reversals, making the body speed the dominant contributor to changes in relative airflow velocity v.

### 3.6. Body oscillations operate at a certain phase relative to the flapping-wing motion to reduce the aerodynamic power requirements

**Figure 8.**
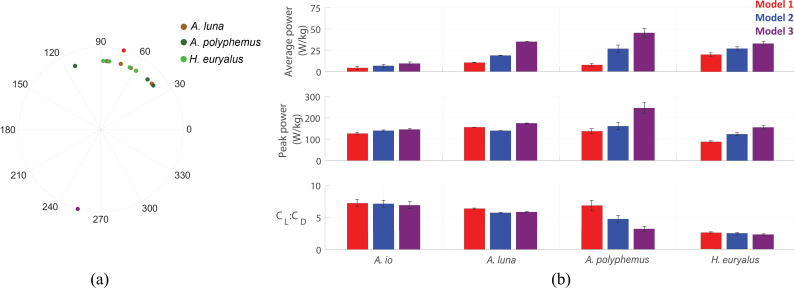
(a) Scatter plot shows phase differences between body pitch angle χ and wing sweep angle ϕ. Red and purple markers on the circumference show phase differences corresponding to Models 1 and 3, respectively, of the representative wingstroke of *H. euryalus*. Each of the other markers shows a single measured wingstroke of silk moths *A. luna*, *A. polyphemus* and *H. euryalus*. (b) Bar plots showing the wingstroke-averaged and peak power dissipated in drag and the lift-to-drag ratio of the four silk moth species *A. io*, *A. luna*, *A. polyphemus* and *H. euryalus*. Three colours of bar graphs correspond to the Models 1 (red), 2 (blue) and 3 (purple) of the body kinematic configurations. Error bars represent the standard error over the 10 trim search trials.

In our data, the waveforms of body pitch angle (χ) and forward (u) and vertical (w) velocity components maintained a consistent shape throughout the wingstroke relative to wing kinematic angles (figure 1). This may indicate a consistent phase relationship between wing and body kinematics. To investigate this possibility, we calculated the phase difference between χ and the wing sweep angle ϕ across the 10 wingstrokes of the three silk moth species with large χ oscillations (*A.luna*., *A.polyphemus*. and *H.euryalus*.) and scattered the values on a polar plot shown in figure 8a. We found that the phase difference remains within a narrow range ($30^{\circ }$–$120^{\circ }$) and spans only about $90^{\circ }$, hinting at potential benefits in flight performance from this preferred phase difference. To explore this further, we used another model of body kinematic configurations: Model 3. In this model, we phase-shifted the body kinematics $180^{\circ }$ relative to the wing kinematics (purple curves in figure 2) and calculated the aerodynamics (purple curves in figures 4, 6 and 7). While such independent decoupling of χ and ϕ is unlikely to be fully realizable in the actual animal, at least without significant inertial reconfiguration, it serves as a useful comparison for the importance of this phase relationship.

We found that the mean and peak aerodynamic force values for Model 3 are greater than those for both Models 1 and 2. Furthermore, Model 3 loses the feature of a small wing inclination angle ($\alpha_{h}$) variation during each mid-halfstroke in Model 1, which held the aerodynamic force in more functionally usable directions of weight support and forward thrust. Model 3 rather generates considerable backward and downward forces during the downstroke and upstroke, respectively. This reduces the effectiveness of the silk moth flight strategy, in which the downstroke provides weight support and the upstroke generates forward thrust. In addition, Model 3 also leads to higher angles of attack ($\alpha_{v}$), particularly during the middle part of the wingstroke (figure 6), where most of the aerodynamic force is generated. This results in a larger drag and, thus, less efficient flight.

The efficiency of flight can also be quantified through aerodynamic power and lift-to-drag ratio calculations. Because silk moth *A.io*. did not experience a considerable magnitude of body oscillations, we found that, except *A.io*., all the other three silk moth species generally showed a large increase in both mean and peak aerodynamic power in Models 2 and 3 compared with Model 1, as shown in figure 8b. Interestingly, the power contribution of the rotational and added-mass force components lowered the mean power because they provided negative power in some parts of the wingstroke. The increase in the mean power requirement for Model 2 ranges from 1.4 times for *H.euryalus*. to 3.8 times for *A.polyphemus*., and for Model 3 it ranges from 1.6 times for *H.euryalus*. to 3.5 times for *A.polyphemus*. Similarly, the increase in the peak aerodynamic power for Model 2 ranges from 1.2 times for *A.polyphemus*. to 1.4 times for *H.euryalus*., and for Model 3 it ranges from 1.1 times for *A.luna*. to 1.8 times for *A.polyphemus*. However, there was a slight decrease in the peak power of *A.luna*. from Model 1 to Model 2. Another important aerodynamic performance metric, the lift-to-drag ratio shown in figure 8b, is a measure of aerodynamic efficiency and is calculated as the ratio between the wingstroke-averaged lift and drag coefficients. In silk moths *A.luna*. and *A.polyphemus*., the lift-to-drag ratio is notably higher for Model 1. This further emphasizes the role of body oscillations and the preference for operating within a certain range of phase difference to improve aerodynamic efficiency.

## 4. Discussion

### 4.1. Body oscillations are associated with wingbeat frequency and wing loading

As established in some butterfly studies [7,8], body oscillations are a consequence of the coupling between wing flapping and body dynamics. Due to wing–body coupling dynamics, all flapping insects experience a degree of body oscillations at wingbeat frequencies. However, in butterflies and silk moths, these oscillations are large and pronounced. Amplitudes of 25−27${,}^{\circ }$ and mean values of 70−80${,}^{\circ }$ are typical of forward flight (less than 1 m s${,}^{-1}$ flight speed) in butterflies *Kallima inachus* [30]. In this study, we observed amplitudes of up to about 40° with a mean value of about 20° in silk moths *A. polyphemus* flying forward at 2−3 m s${,}^{-1}$ with up to 1 m s${,}^{-1}$ variation in vertical speed per wingstroke.

Compared with insects of similar body size and mass, butterflies and silk moths have low wingbeat frequencies but large wings and flapping amplitudes to generate sufficient force for flight. Because the oscillation amplitude increases with the flapping period and varies inversely with wing loading, it is expected that the body oscillation amplitude is larger in butterflies and silk moths. Wing flapping excites the pitching dynamics of the body as a result of a time-varying pitching moment generated by both the aerodynamic and inertial forces of wing flapping. A flapping flier with larger wings generates larger peak forces and, thus, a larger peak pitching moment. A flier with a smaller body mass has a smaller body moment of inertia, and thus its body is prone to experiencing larger pitch accelerations. These larger peak accelerations, when applied periodically through lower wingbeat frequencies (and thus longer periods), make the body pitch angle oscillate at larger amplitudes. As we show in our results, the product of the wingbeat frequency and wing loading of silk moths is negatively correlated with the amplitude of body pitch oscillations. Thus, silk moths with slower and larger wings and smaller body masses exhibit larger magnitudes of body oscillations. Although body pitch oscillations can be explained using a passive mechanism, the role of active abdominal flexion in partially inducing and controlling body pitch oscillations cannot be ignored due to the evidence for active abdominal flexion in moths and butterflies [8,11,31].

### 4.2. Wing inertial force alone cannot explain body oscillations

While silk moths and butterflies are probably convergent in their body oscillations because the bombycoid moths ancestral to silk moths probably had wing shapes more similar to hawkmoths (Sphingidae), the mechanism for producing them seems different. In silk moths, we found that both aerodynamic and inertial forces (figure 4) contribute roughly equally to the body pitch and vertical velocity oscillations, as opposed to the cases of birds and butterflies, where the wing inertial and aerodynamic forces, respectively, drive body oscillations [7,14]. Silk moth wings are thin membranes covered in tiny scales made of chitin. Unlike birds, they lack bones or muscles within the wing itself, and thus their wings are much less dense than bird wings. This implies that for wings of equal area and similar movement, a silk moth wing would impart a smaller inertial force but equal aerodynamic force on the body as compared with a bird wing. Thus, the wing inertial force in silk moths contributes comparably with the effects of the aerodynamic force on the within-wingstroke dynamics of flight.

### 4.3. Body oscillations reduce the power requirements of flight by redirecting aerodynamic force and reducing drag

In steady forward flight, silk moths (in this study) and butterflies pitch nose down during the downstroke and nose up during the upstroke [31,32]. This contributes to a time-varying stroke plane that remains steep during the downstroke and shallow during the upstroke [31]. This allows these fliers to redirect the aerodynamic force in functionally usable directions [32]. Specifically, a shallower stroke plane supports weight during the downstroke, and a steeper stroke plane generates forward thrust during the upstroke by changing the jet flow direction [30,31]. Moreover, fliers can also control the direction of flight by controlling the direction of this jet flow. Thus, they can transition between forward and vertical flight by modulating the mean and amplitude of the body pitch oscillations [30]. However, it was not well known how the body oscillations affect the inclination angle of the wing, and thus redirect the aerodynamic force into usable directions.

Although reducing the power requirement of flight is beneficial, reducing drag in fliers like silk moths that use larger stroke-plane angles during the downstroke and smaller stroke-plane angles during the upstroke can potentially reduce weight support and forward thrust due to a reduced overall aerodynamic force. However, body pitch (χ) oscillations can compensate for this effect by keeping the wings inclined at appropriate angles during each half-stroke. Using our blade-element model, we found that oscillations in χ influence wing motion in a way that redirects the aerodynamic force vector, enhancing weight support during the downstroke and forward thrust during the upstroke. In the first half of the downstroke, a shallower body angle enforces a smaller $\alpha_{\text{h}}$, and hence directs the aerodynamic force vector more vertically upward. Thus, most of the aerodynamic force during the downstroke provides weight support rather than applying backward thrust on the body. For our species-averaged kinematics of silk moths *A. luna* and *A. polyphemus*, $\alpha_{h}$ dips further and even stays negative for most of the downstroke duration. This makes the horizontal components of the aerodynamic force directed forward, hence, providing forward thrust even during the downstroke. In the latter half of the upstroke, a larger $\alpha_{h}$ (greater than $50^{\circ }$) directs the aerodynamic force more horizontally forward, which means most of the aerodynamic force generated during this half-stroke is used in propelling the body forward rather than pushing it downward. This also enhances forward thrust and the overall weight support over the entire wingstroke.

Body oscillations have probably independently evolved in both silk moths and butterflies and have convergent aerodynamic consequences. Butterflies have also been shown to generate most of their weight support during the downstroke and propel themselves forward during the upstroke [30]. However, in the current study, we found that considerable forward propulsion can also be generated during the downstroke if the body tilts further nose-down, causing the wing surface to incline below the absolute horizontal plane.

Compared with other insects of similar body size and mass, butterflies and silk moths save power by using low wingbeat frequencies [4]. However, flapping larger wings with a fraction of power spent on exciting body oscillations increases the power requirements for flight. For both hover and forward-climbing trajectories of Monarch butterflies, passive body oscillation arising from wing–body coupling results in a reduction in energy and power consumption [8]. In our study, we found that body oscillations in silk moths, particularly oscillations in body speed, can help maintain a low angle of attack $\alpha_{v}$ of the wing. This is achieved by body pitch oscillations operating at an appropriate phase relative to the flapping-wing motion, such that the incoming airflow is incident at a smaller angle on the wing, thus maintaining a smaller $\alpha_{v}$ particularly during the downstroke. This results in smaller drag and thus reduces the aerodynamic power requirements of flight. This hints at a potential role of wing–body coupling in improving flight control and aerodynamic efficiency in silk moths.

Although our model can directly separate the contributions of wing and body kinematics to aerodynamic force and power, these factors are coupled in the actual animal. Indeed, the body oscillations are probably created in part by the aerodynamic forces themselves and then alter the aerodynamic forces of the wing through their effects on the wing kinematics. Therefore, it is likely that the silk moth may not be able to fully suppress body oscillations, even if it attempted to do so. In addition to wing–body coupling, active abdominal flexions may also contribute to body pitch oscillations. A study on monarch butterflies found that oscillatory abdominal flexions, which are more pronounced in butterflies, increase the magnitude of body oscillations [8]. This results in a reduction in mean flight power requirements by up to 6%, providing further evidence for reduced power requirements due to body oscillations [8]. Active abdominal flexion in a hawkmoth and some butterfly species has an active role in flight stabilization [11,31,33,34]. Active articulation of the thoracic-abdominal joint redirects the aerodynamic force vector for effective flight control [11]. This force redirection is similar to what we refer to as force redirection arising from the wing rotation due to body pitch oscillations. Even though we have not explored the effect of this force redirection on flight stability, the body oscillations probably reduce the peak pitching moment over each half-stroke and hence reduce the total pitch impulse the body experiences in each half. This may provide a passive pitch stability mechanism in silk moths during steady forward flight. Most passive stability analyses in the past have focused on fliers with high wingbeat frequencies that do not display passive body oscillations. Thus, our work also lays the foundation for a future study in silk moths to directly test the role of body pitch oscillations in pitch stability.

## 5. Conclusion

Many silk moths with low wingbeat frequencies and larger wings exhibit erratic flight behaviour. Their bodies bob and pitch in a periodic fashion during steady forward flight. These body oscillations are probably coupled to their flapping-wing motion, with important implications for flight control and performance. To investigate the forces responsible for body oscillations and their aerodynamic consequences on flight performance, we recorded three-dimensional forward flight kinematics of four silk moth species flying in a wind tunnel. After extracting kinematic measurements and making species-averaged aerodynamic fits, we calculated the aerodynamic forces and power using a quasi-steady blade-element method to analyse the dynamics and performance of flight. Given the observed kinematic patterns, we found that the inertial and aerodynamic forces of our silk moth specimens were equally dominant in generating body oscillations in linear velocity and body pitch angle. The oscillations in body pitch angle are associated with the wingbeat frequency and wing loading of silk moths and are probably coupled with the movement of flapping wings. This coupling was evident from a small range of phase difference between body pitch angle and wing sweep angle, where flight power requirements are reduced by redirecting the aerodynamic force into functionally usable directions of weight support and forward thrust while lowering the drag force on the wings. The oscillatory body pitch relative to periodic wing flapping occurs at a phase that maintains a lower angle of attack and orients the wings to redirect aerodynamic forces, thereby enhancing both upward and forward force production. This reduces the total aerodynamic force requirements by reducing the drag force without compromising weight support and forward thrust. Body oscillations may be ecologically important for silk moths in two key ways. First, they manifest as erratic flight behaviour, which can aid in evading predators. Second, they reduce the aerodynamic power requirements of flight, which is a crucial advantage given that adult silk moths do not feed and rely on limited energy reserves.

## Data Availability

Data and code are available from Dryad Digital Repository [35]. Supplementary material is available online [36].
